# Analysis of the atrophic mandible rehabilitated with fixed total prosthesis on mono or bicortical implants

**DOI:** 10.1590/0103-6440202405621

**Published:** 2024-06-24

**Authors:** Fabricia Carla Martins Bezerra Garutti, Roberto Brunow Lehmann, Ivan Onone Gialain, Fernando Fusari Bento de Lima

**Affiliations:** 1 Graduate Program in Health Sciences, Medical Schoolof the Federal University of Mato Grosso, Cuiabá/MT- Brazil; 2 Mechanical Engineering Department, Volta Redonda School of Industrial Metallurgical Engineering, Federal Fluminense University, Volta Redonda/RJ- Brazil; 3 Graduate Program in Integrated Dental SciencesUniversidade de Cuiabá, Cuiabá/MT- Brazil

**Keywords:** Finite elements, bone biomechanics, bicorticalized implants, monocorticalized implants.

## Abstract

Rehabilitation of edentulous atrophic mandibles involves the placement of implants in the anterior segment of the mandible. The primary stability of these implants can be improved using the base of the mandible as complementary anchorage (bicorticalization). This study aimed to analyze the biomechanics of atrophic mandibles rehabilitated with monocortical or bicortical implants. Two three-dimensional virtual models of edentulous mandibles with severe atrophy were prepared. Four monocortical implants were placed in one model (McMM), and four bicortical implants were placed in the other (BcMM). An implant-supported total prosthesis was prepared for each model. Then, a total axial load of 600 N was applied to the posterior teeth, and its effects on the models were analyzed using finite element analysis. The highest compressive stresses were concentrated in the cervical region of the implants in the McMM (-32.562 Mpa); in the BcMM, compressive stresses were distributed in the upper and lower cortex of the mandible, with increased compressive stresses at the distal implants (-63.792 Mpa). Thus, we conclude that axial loading forces are more uniformly distributed in the peri-implant bone when using monocortical implants and concentrated in the apical and cervical regions of the peri-implant bone when using bicortical implants.

## Resumo

A instalação de implantes no segmento anterior da mandíbula, é um tratamento utilizado para reabilitação de mandíbulas atróficas. Para melhorar a estabilidade primária desses implantes, a base da mandíbula pode ser usada como ancoragem complementar (bicorticalização). Este estudo objetiva analisar a biomecânica de mandíbulas atróficas, reabilitadas com prótese sobre implantes monocorticalizados ou bicorticalizados. Para isso foram confeccionados dois modelos tridimensionais de mandíbula desdentada e com atrofia severa. Em um deles foram instalados 4 implantes monocorticalizados (McMM), enquanto no segundo foram instalados 4 implantes bicorticalizados (BcMM); foi modelada uma prótese total implantossuportada sobre cada modelo e aplicada uma carga axial total de 600N, distribuída nos dentes posteriores. Os modelos foram submetidos à análise de elementos finitos. Os resultados demonstraram que as maiores tensões de compressão se concentraram na região cervical dos implantes no McMM, (-32,562Mpa); já no BcMM, as tensões de compressão foram observadas nas corticais superior e inferior da mandíbula e aumento das tensões de compressão nos implantes distais (-63,792 Mpa). Com isso, concluímos as forças de carregamento axial apresentam-se melhor distribuídas pela estrutura óssea peri-implantar, em implantes monocorticalizados. e as tensões sobre o tecido ósseo, no BcMM, ocorrem nas regiões que circundam as regiões apicais e cervicais do implante.

## Introduction

Oral health is critical for maintaining an individual's quality of life, while tooth loss has been a public health problem and, for many years, rehabilitation with dental or mucosupported prostheses was seen as the only way to treat and restore the health of these patients ^1^. With the advent of implant dentistry and its high success rates, the use of implants has been encouraged to treat complete, partial, and single edentulism.

Oral rehabilitation using osseointegrated implants requires sufficient alveolar bone for primary implant stability. However, a decrease in alveolar bone volume (due to physiological resorption, trauma, or pathology)^2^ results in considerable changes, hindering the primary stability required for implant osseointegration^3^.

Thus, dental implants and surgical techniques have been developed and modified to ensure safe and predictable functional and esthetic results^3^ while reducing the need for grafting, making the treatments less invasive and time-consuming^4^.

Morphologically, the basal cortical mandible´s bone, in the anterior segment, is thicker than the buccal and lingual cortical plates ^5^, so bicorticalization promotes greater primary stability, due to greater contact between the implant and this basal bone, and can be applied in various regions of the oral cavity where the bone quality indicates poor prognoses for implant stabilization ^2^ ^,^ ^6^; however, they are associated with higher rates of implant fractures than monocortical implants ^7^, and has also been associated with the computer-guided installation of implants in atrophic jaws ^6^.

Some bicorticalization protocols have been evaluated, among the variations studied, are the three-dimensional positioning of implants in atrophic jaws ^8^, implant design ^9^, and mandibular biomechanical aspects ^10^.

Recently, finite element analysis (FEA) has been widely used to simulate and investigate the effects of occlusal loads on dental implants of various lengths and peri-implant bone ^11^. It has been used to study models of implant-supported total prostheses ^12^, fixed partial dentures ^13^, and bone biomechanics ^10^ ^,^ ^11^.

However, literature on bone biomechanics of mandibular alveolar ridges with severe resorption is scarce; thus, this study aimed to analyze the biomechanics of severely atrophied edentulous mandible rehabilitated with implant-supported total prostheses on monocortical or bicortical implants.

## Methodology

This study involved only virtual simulations and thus did not require ethics committee approval.

**Figure 1:**
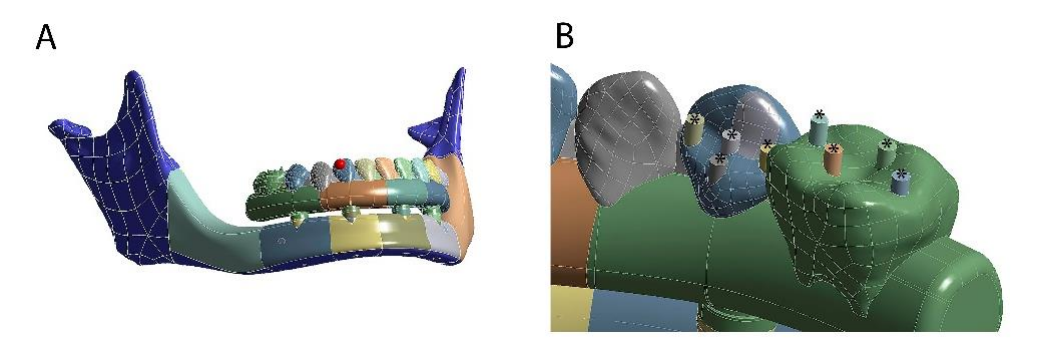
Model of a mandible with severe resorption of the alveolar process. A) General view of the mandible model, with implants (intraosseous), prosthetic components, and implant-supported prosthesis. B) Approximate view of the occlusal surface of the elements with the load applicators (asterisk)

Two identical three-dimensional atrophic mandible models were generated from a non-atrophic edentulous mandible model, belonging to the biobank of models at the Fluminense Federal University. This mandible was remodeled using the Ansys software, version 14, where the original dimensions of the alveolar process were altered to present atrophy of the alveolar process (Figure 1).

The models were considered atrophic when the height between the base of the mandible and the apex of the alveolar process reached only 10mm, this being the final height of the region between the mental foramina.

**Figure 2:**
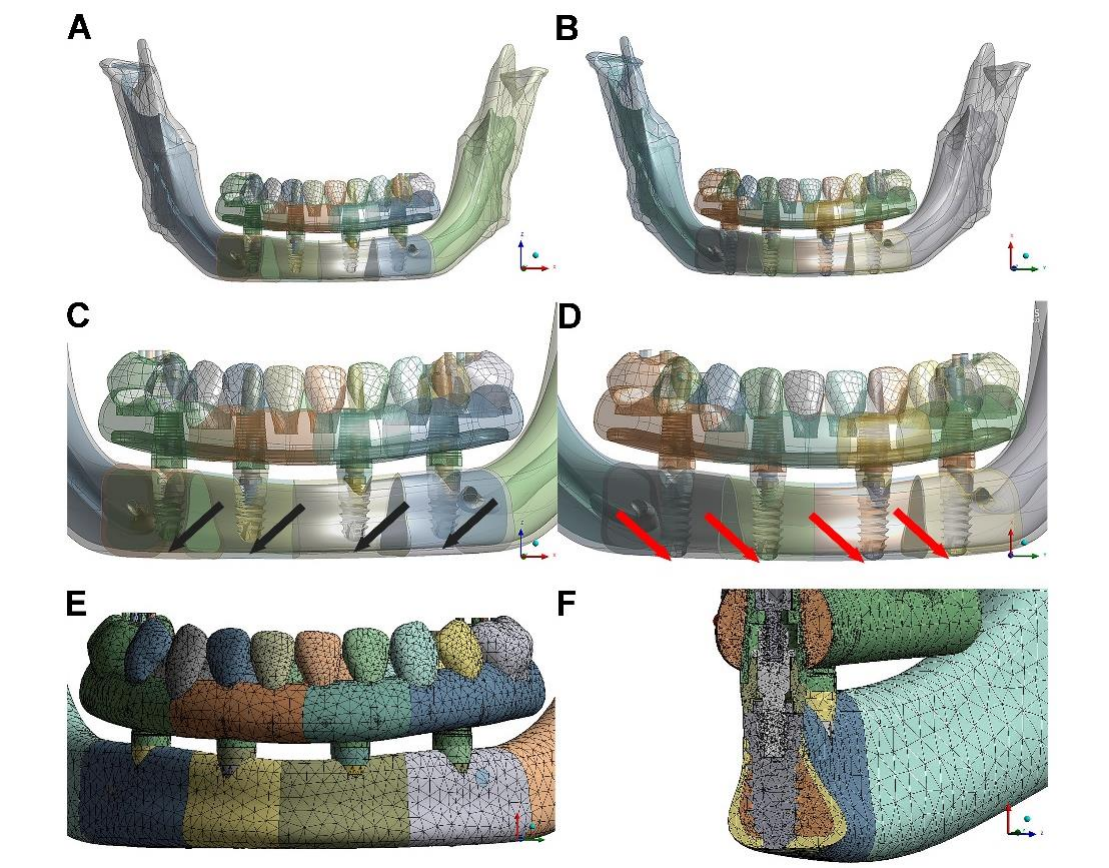
Monocortical and bicortical implant models. A) Anterior view of the McMM; B) Anterior view of the BcMM; C) Approximate view of A, the McMM with space between the apex of the implant and the inferior cortical bone of the mandibular base (black arrows); D) Approximate view of B, the BcMM without space between the apex of the implant and the inferior cortical bone of the mandibular base (red arrows); E) Finite element mesh generated for analysis in the region between the mental foramina and in the prosthesis. F) Parasagittal section of the right anterior implant (element 42), illustrating the finite element mesh generated in the region. McMM, monocorticalized mandible model; BcMM, bicorticalized mandible model.

A total of four conical external hexagon implants (INP - São Paulo, Brazil) were symmetrically placed between the mental foramina of each model. In one model, implants of 4.1 mm diameter and 8 mm length were placed such that only the cervical portion of the implant contacted the cortical bone; this model was called the monocortical mandible model (McMM) (Figure 2).

In the second model, implants of 4.1 mm diameter and 10 mm length were placed such that their cervical and apical portions contacted the cortical bone (perforating it); this model was called the bicortical mandible model (BcMM) (Figure 2).

In both situations, conical mini-pillar models (INP - São Paulo, Brazil) were installed with through-bolts, and an implant-supported total prosthesis was designed. To reduce the distal length of the prosthesis, it included only 10 teeth: all anterior teeth, first premolars, and first molars.

To optimize the application of load on the occlusal surface of the premolars and molars, four load applicators of 1-mm diameter were used for each tooth ^14^ (Figure 1).

A finite element mesh was generated (Figure 2), and a sensitivity study was performed. The element size, element growth factor, and presence or absence of “mid-nodes,” among other possible options, were defined according to the physical characteristics of the volume.

A maximum element size of 0.8 mm was considered. Virtual topology was designed to improve element distribution. The relative absence of movement between the bodies and perfect osseointegration were considered (“bonded” contact was selected at the bone-implant interface).

The following simplifying assumptions were adopted. The materials were considered homogeneous, isotropic, and linearly elastic ^15^. The implants, mini-pillars, and prosthetic screws were modeled in titanium ^12^. The properties of cortical and trabecular bone were set as those described previously ^12^. The metallic infrastructure was modeled in a nickel-chromium alloy ^12^ and the teeth and covering of the infrastructure were in acrylic resin ^12^. Finally, the access channels to the prosthesis retention screws were filled with composite resin (Filtek Supreme) ^16^. The characteristics of the materials used in this study are presented in Box 1.

**Box 1:**
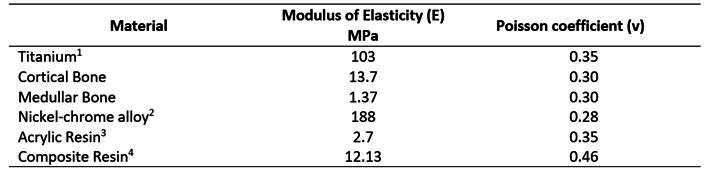
Mechanical properties of the materials used

1- implants, mini-pillars, and prosthetic retention screws; 2- metallic infrastructure; 3- teeth and covering of the metallic infrastructure; and 4- used to cover the access channels to the prosthetic retention screws.

To evaluate the stress transmitted to the cortical and medullary bones, the workbench environment of ANSYS^TM^, version 14, was used. A load of 37.5 N was applied to each load applicator, resulting in a 150 N load on each posterior tooth and 600 N on the complete model. The modulus of elasticity and Poisson's ratio used in this study are presented in Box 1.

A preconditioned conjugate gradient (PCG) solver was applied for the models, which had more than 50,000 elements.

A color gradient was used to present the stress distribution at each modeled component (independent volumes), allowing individual analysis of each prosthetic component. The maximum stress at each component was compared with the yield strength of the corresponding material.

The principal stress criterion was used for brittle materials, such as resin and bone. The maximum negative values, indicative of compression, were considered in the principal stress analysis.

For movement restriction, the posterior portion and base of the mandible ^17^ were chosen to confer stability to the model, disregarding the proximity of the bone to the implant.

## Results

The number of nodes and elements generated in the models used in this study are presented in Table 1.

**Table 1:** Number of elements and nodes generated in the finite element mesh of the models

	McMM	BcMM
Elements	345.204	405.299
Nodes	608.891	633.886

McMM, monocorticalized mandible model; BcMM, bicorticalized mandible model

The maximum principal stress was analyzed only for bone owing to its critical tensile and compression characteristics.

**Figure 3:**
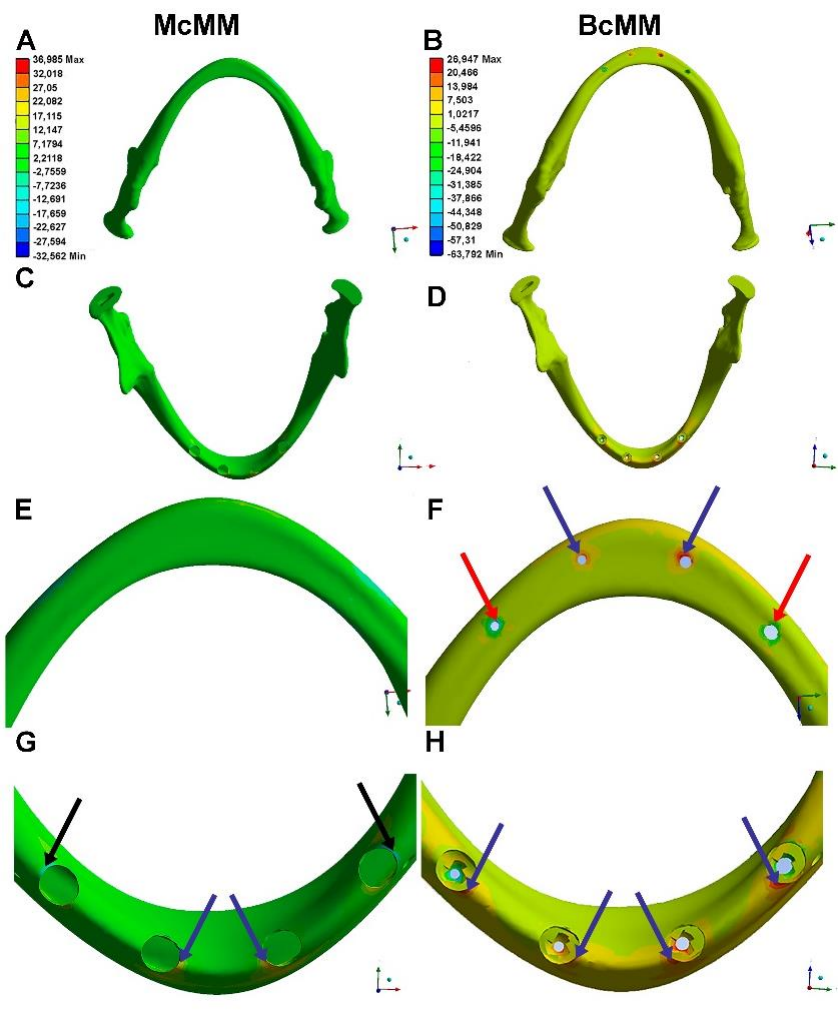
Finite element analysis of the upper and lower cortex of the mandible in the McMM (left) and BcMM (right). A and B) Results of the finite element analysis in a lower view. C and D) Results of finite element analysis in an upper view. E and F) Approximate view of A and B, respectively. G and H) Approximate view of C and D, respectively. The scale presented in image A is the same as that used for images C, E, and G. The scale in image B is the same as that used for images D, F, and H. Black arrows indicate the points of higher compressive stress in McMM. Red arrows indicate the points of higher compression in BcMM. Blue arrows indicate the main traction points in the MCMM and BcMM models. McMM, monocorticalized mandible model; BcMM, bicorticalized mandible model.

In the McMM, the compressive forces following the application of occlusal load on the posterior teeth (premolars and molars) were concentrated in the cervical region of the implants near the cortical portion of the residual alveolar ridge, with a minimum stress of -32.562 Mpa (Figure 3G). Compressive stresses (negative values) were observed only at the distal implants, while no such forces were present in the lower cortical region (Figure 3A and 3E).

**Figure 4:**
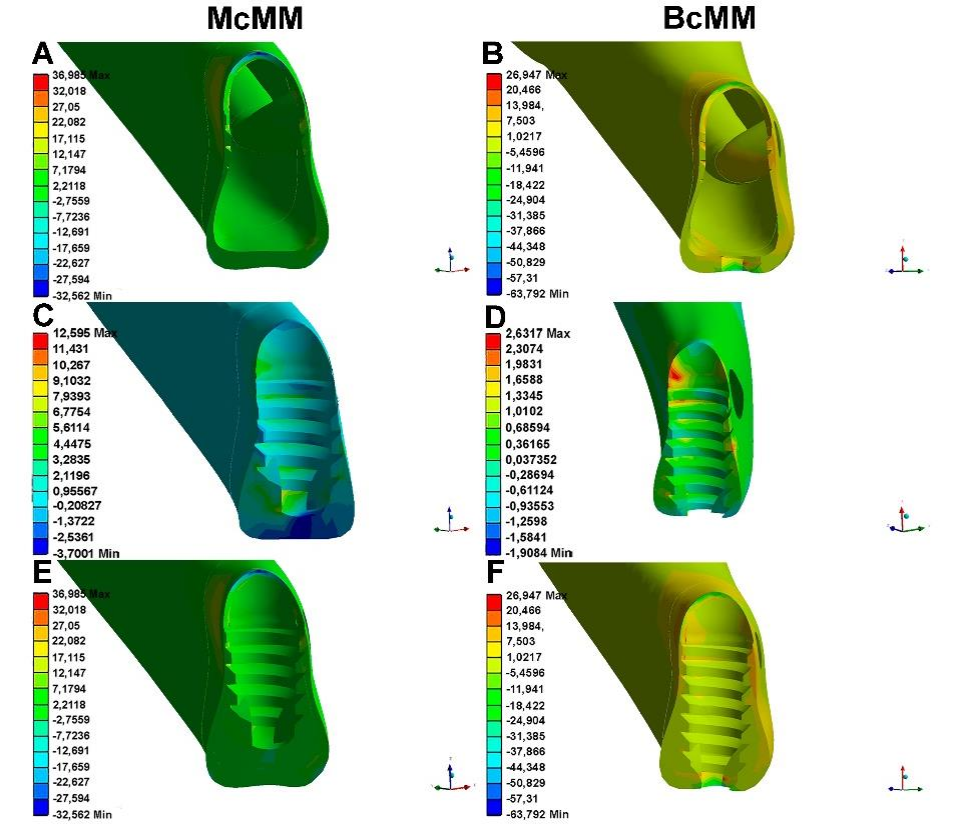
Longitudinal section of a left distal implant from the McMM (left) and BcMM (right). A and B) Results of finite element analysis on cortical bone. C and D) Results of the finite element analysis on medullary bone. E and F) Results of the finite element analysis on cortical-medullary bone. McMM, monocorticalized mandible model; BcMM, bicorticalized mandible model

In the BcMM, the post-loading compressive stresses were the highest in the lower cortical regions (Figure 3F). Furthermore, the compressive stresses at the distal implants were higher in the BcMM than in the McMM. However, the compressive stresses were more intense in the mandibular inferior cortical region, especially in the distal implants (-63.792 Mpa) (Figures 3F and 4).

Tensile stress was observed in the mesial implants of the McMM and BcMM models, however in the BcMM model this stress was observed in the superior and basal corticals of the mandible (Figures 3F and 3H), while in the McMM, at a lower intensity only in the superior cortical bone ( Figure 3G).

Examination of a longitudinal section of a left distal implant in the McMM revealed that the forces were well distributed, especially in the medullary bone, as it shows less resistance to the applied load and greater deformation. Compression forces were observed in the upper cortical bone near the implant neck (Figure 4).

The minimum principal stress values in the cortical and medullary bone are presented in Table 2.

**Table 2:** Minimum values of principal stress on the cortical and medullary bone in both models

	Principal compression (MPa)	
	Cortical bone	Medullary bone
McMM	32.562	3.7001
BcMM	63.792	1.9084

McMM, monocorticalized mandible model; BcMM, bicorticalized mandible model

## Discussion

The stress, when concentrated in the cortical bone, promotes bone resorption near the implant platform ^18^. Compressive and/or tensile stresses, when exerted above the threshold tolerated by the cortical bone tissue (167 and 121 MPa, respectively) ^19^, may have deleterious effects, including loss of bone in the cervical regions as the cortical bone exhibits little plasticity and high rigidity. Our results demonstrate that compressive stresses within the recommended range did not promote bone destruction in both models.

The use of short implants (length ≤ 6 mm) has been suggested in cases of reduced alveolar bone height; however, they are associated with a lack of predictability ^13^. Furthermore, it isn’t recommended their use in conditions of a combination of low bone quality and high occlusal forces for biomechanical reasons ^20^ ^,^ ^21^. These implants cannot help correct the unfavorable biomechanical aspects and bone discrepancies; therefore, their distance from the occlusal plane, length, and forces should be appropriately evaluated.

With advances in surgical techniques, implant surface treatment, and the concept of primary stability, the use of short implants has become a viable option for atrophic mandibles ^22^ ^,^ ^23^ and atrophic maxilla’s ^24^. Furthermore, the loss rate for short implants is considered only 20% ^25^. Through the results of the present study, where the McMM showed better distribution of forces and smaller regions of bone compression concentrated in the cervical region, we can infer that dental rehabilitation on alveolar processes with severe atrophy, associated with shorter monocorticalized implants, becomes a viable alternative and a treatment option for these patients.

Bicortical implants have previously shown low stress levels in the cortical bone around the implant neck ^26^. In contrast, the present FEA showed a significant increase in the maximum stress levels both in the upper and lower cortical bone, especially in the distal implants of the BcMM.

Bicorticalization is highly predictable with high implant survival rates ^27^ and favors primary stability ^28^; therefore, is a favorable option when considering the immediate loading of osseointegrated implants ^29^. The present results indicate the possibility of bone tissue remodeling in the cervical and apical regions of the BcMM, which may be favorable for primary implant stability. This aspect should be considered during implant planning.

Another consideration regarding bicorticalization refers to maintaining the integrity of the mandible and resistance to fracture when occlusal loads are applied. The installation of bicortical implants, in the posterior region of the mandible, did not demonstrate a significant reduction in their resistance ^30^. Our results are in agreement with those obtained in the posterior region of the mandible, as they demonstrate greater compression and traction stress in the basal cortex of the BcMM, however, this stress is fully bearable by the bone structure simulated in this study, providing security to the surgeon who opts for bicorticalization, regarding jaw strength.

Biologically, a factor to be considered when using bicorticalization in the anterior segment of the mandible refers to the large amount of cortical bone that surrounds all the implant faces in this region^5^; considering the low vascularization of cortical bone tissue and its slow metabolism, it´s may be associated with higher rates of bone loss implants of this technique ^7^.

Prosthetic factors can also influence bone biomechanics in mono- or bicorticalized study models. The use of flexible connectors reduces bone tension around monocorticalized implants ^31^, these results can improve bone biomechanics in areas of bicorticalization, and however, this reduction was not observed in this study because it was in rigid connectors. On the other hand, the design of the implant does not seem to influence bone biomechanics in the rehabilitation of implant-supported edentulous mandibles ^9^.

Our results also confirm the findings, which state that the biomechanical behavior of shorter monocorticalized implants, in different positions and different angulations, present less stress when loaded with unilateral forces of 100N ^8^.

It should be emphasized that this simulation study considered complete osseointegration of the implants. The implants are loaded before osseogration has occurred in cases of immediate loading. Thus, further studies are needed to better understand the utility of the bicorticalization technique in cases of immediate loading after implant placement.

Stress analysis using FEA on osseointegrated implants of the same diameter with monocortical and bicortical anchorage and an axial load of 100 N concentrated on half the radius of the prosthesis indicated lower stresses in the cortical bone around the bicortical implants than those around monocortical implants ^7^. Thus, it can be inferred that the load applied to the models used in the FEA and their distribution may have fundamental importance in bone biomechanics, as the results of this study differ from those presented here.

A retrospective study on the influence of implant anchorage type showed different results. The study showed that the failure rate of implants with bicortical anchorage was four times higher than that of monocortical implants ^32^. In this study, the bicortical implant use was associated with the highest compressive stress at the upper and lower cortex of the mandible; depending on the intensity, this stress could have deleterious effects on implant stability if exerted over a long term ^7^.

The success and longevity of implants are closely dependent on mechanical factors and stresses exceeding physiological limits have been suggested as the major causes of early peri-implant bone loss ^7^ ^,^ ^19^. This underscores the importance of this study and corroborates the finding of bone stress in the peri-implant regions of both models, which may subsequently induce peri-implant remodeling in the clinical scenario.

FEA is an important tool used for biomechanical evaluations across several dental and medical disciplines. However, it has some limitations. Therefore, further virtual simulation, retrospective, and randomized clinical studies in different clinical scenarios are necessary to elucidate the behavior of peri-implant bone and increase the predictability of rehabilitation treatments for patients with edentulous and severely resorbed alveolar processes.

## Conclusion

Within the limitations of a virtual simulation study, we can conclude that the axial loading forces are better distributed throughout the peri-implant bone structure, demonstrating smaller areas of compressive stress on the bone structure when using monocortical implants. Conversely, when using bicortical implants, the stresses are distributed in the upper and lower cortices of the mandible, that is, in the bone surrounding the apical and cervical parts of the implant.
